# Draft Genome Sequence of Methanobacterium paludis IBT-C12, Recovered from Sediments of the Apatlaco River, Mexico

**DOI:** 10.1128/mra.00906-21

**Published:** 2022-02-03

**Authors:** Erick Alejandro Sánchez-Salazar, Lizbeth Hernández-Jaimes, Luz Breton-Deval, Ayixon Sánchez-Reyes

**Affiliations:** a Universidad Autónoma Metropolitana, Unidad Iztapalapa, Mexico City, Mexico; b Centro de Investigación en Dinámica Celular, Universidad Autónoma del Estado de Morelos, Cuernavaca, Morelos, Mexico; c Cátedras Conacyt-Instituto de Biotecnología, Universidad Nacional Autónoma de México, Cuernavaca, Morelos, Mexico; Montana State University

## Abstract

Methanobacterium paludis is a hydrogenotrophic archaea first described in 2014 and isolated from a peatland area. So far, there is only one sequenced genome of this taxon. Here, we report the draft genome sequence of *M. paludis* IBT-C12, a metagenome-assembled genome (MAG) from sediments in the Apatlaco River, Mexico.

## ANNOUNCEMENT

Methanobacterium paludis is an anaerobic archaeon initially described in 2014 and isolated from a peatland area. It uses a hydrogenotrophic pathway to reduce CO_2_ to methane on methanogenesis (1). There is little information on the genomic complement of this methanogenic species, with only one available assembly for the type strain, SWAN1, on the National Center for Biotechnology Information (NCBI) (accession number GCA_000214725.1) (2). Other genomic composites would be crucial to gain information on the metabolism, ecology, taxonomy, and other traits of environmental relevance. Here, we report the draft genome sequence of *M. paludis* IBT-C12, a metagenome-assembled genome (MAG) recovered from freshwater sediments in a highly polluted river in Mexico (3, 4). The MAG was isolated from an environmental sample. Full information about the sample nature, collection site, deconvolution processes, and data were reported before (3). Briefly, sediments were collected at 10-cm depth at a rate of 0.5 kg per site and were stored in coolers at 4°C until further processing. Metagenomic DNA was extracted using the DNeasy PowerWater kit (Qiagen, Hilden, Germany) according to the manufacturer’s recommendations. The *M. paludis* IBT-C12 genome was deconvolved from two sets of short reads obtained from freshwater and sediment in the Apatlaco River, Mexico. One read set was acquired from freshwater on a shotgun NextSeq 500 sequencing platform (Illumina, Inc., San Diego, CA) (108,785,988 paired-end reads, 75 bp) and a set of Hi-C reads from sediments (72,007,031 paired-end reads, 150 bp) from a HiSeq 4000 Illumina-compatible sequencing library. The shotgun library was prepared using the TruSeq kit version 2 (Illumina, Inc.) according the manufacturer’s protocol. The ProxiMeta microbiome kit (Phase Genomics, Seattle, WA) was used to prepare the Hi-C library, and the DNA was digested with Sau3AI and MlucI enzymes before proximity ligation. Read quality was assessed and filtered under the Phase Genomics cloud-based bioinformatics portal by using the Fastp tool version 0.23.1 (5, 6). A metagenome assembly was constructed with the shotgun reads (only the NextSeq data were used) using MEGAHIT software version 1.2.9 (7) (size 550,428,717 bp and 746,031 contigs). The Hi-C reads were subsequently mapped with the Burrows-Wheeler Aligner MEM algorithm (BWA-MEM) version 0.7.17 (-5SP and -t 8 options) to create Hi-C-based contact probability maps (8). The binning was performed with ProxiMeta software (5), resulting in 97 MAGs (3). The MAG corresponding to IBT-C12 was identified by comparing genome relatedness indexes (mash distance [9] and average nucleotide identity [ANI] [10, 11]) against a custom database (12). Default parameters were used for all software unless otherwise noted. The total length of the *M. paludis* IBT-C12 genome was 2,696,644 bp, with a G+C content of 35.95%. The number of contigs was 374, with an average shotgun sequencing coverage depth of 16× and an *N*_50_ value of 1,849,042 bp. The completeness and contamination were estimated with miComplete version 1.1.1 (13) at 61.83% and 1.13%, respectively. The genome size was close to that of the reference strain, SWAN1 (2,546,541 bp), so IBT-C12 MAG must be moderately complete.

**FIG 1:**
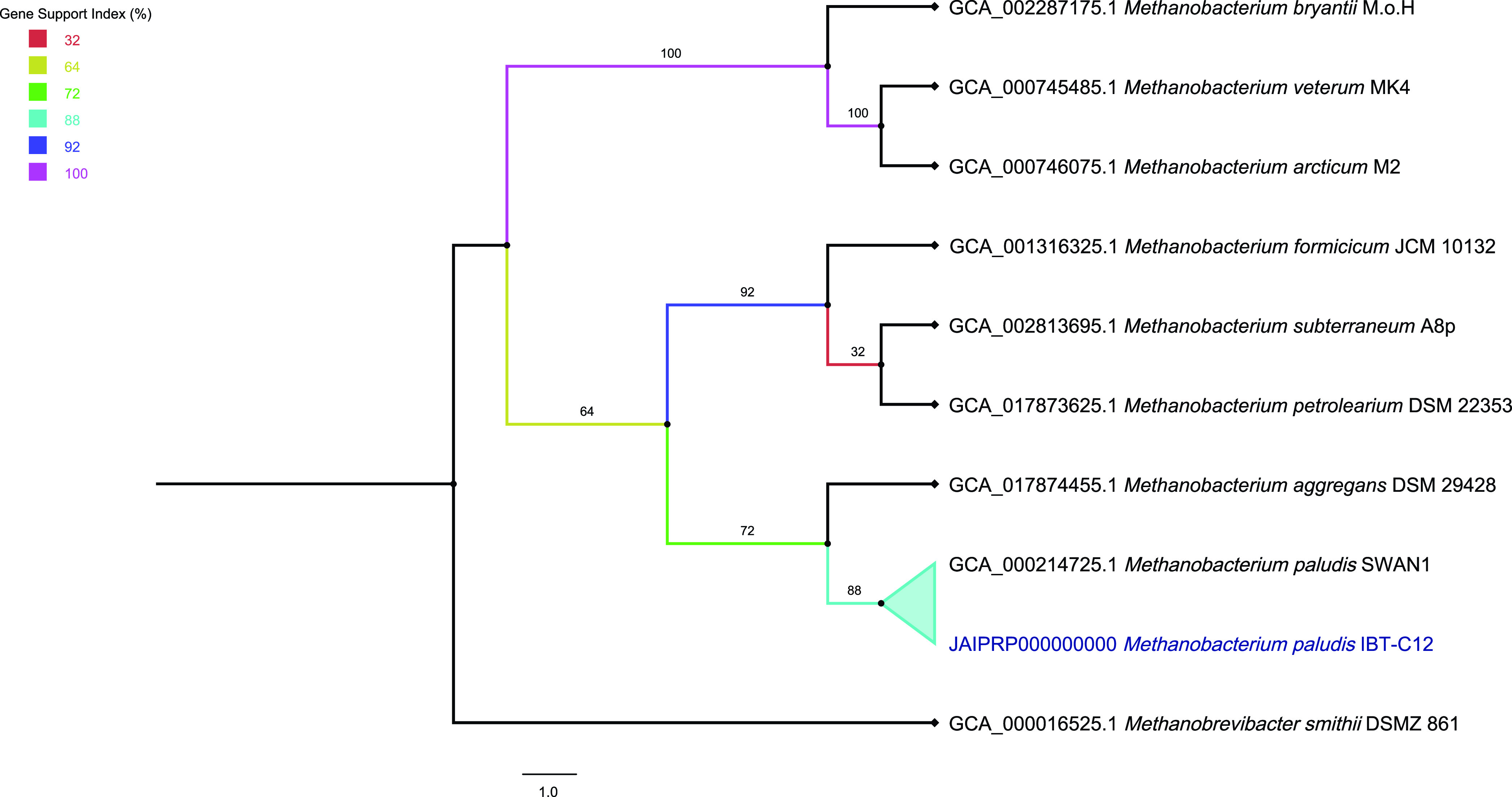
FastTree phylogenomic tree using 25 core genes inferred with the UBCG pipeline (14, 16). The 25 core genes were automatically aligned with MAFFT and concatenated (17). Poorly conserved regions were curated with Gblocks (18). Gene support indices (GSIs) as percentages are shown at branching points. Bars indicate substitution per site.

The IBT-C12 strain is closely related to *M. paludis* SWAN1, as shown by a FastANI result of 94.34% (95.2% with OrthoANI version 0.5.0) and the phylogenomic analysis (Table 1; Fig. 1) (10, 14). The annotation of the MAG sequence was performed with the DFAST service version web with default parameters (15). A total of 2,550 genes were identified, with 2,514 protein-coding sequences and 36 RNA genes. The RNA genes comprised 1 partial 16S rRNA, 2 partial 23S rRNAs, and 33 tRNAs; 2 CRISPR arrays were also identified.

**TABLE 1:** Average nucleotide identity and Mash mutational distance (D) of Methanobacterium paludis IBT-C12 compared with the closest NCBI type strains of the genus *Methanobacterium*

GenBank assembly accession no.	Reference organism	OrthoANI (%)	Mash D
GCA_000214725.1	Methanobacterium paludis SWAN1	95.20	0.05
GCA_017874455.1	Methanobacterium aggregans DSM 29428	78.02	0.22
GCA_002287175.1	Methanobacterium bryantii M.o.H	71.07	0.26
GCA_000745485.1	Methanobacterium veterum MK4	70.64	0.26
GCA_000746075.1	Methanobacterium arcticum M2	70.60	0.26
GCA_001316325.1	Methanobacterium formicicum JCM 10132	69.41	0.30
GCA_017873625.1	Methanobacterium petrolearium DSM 22353	74.38	0.30
GCA_002813695.1	Methanobacterium subterraneum A8p	69.33	1.00
GCA_000016525.1	Methanobrevibacter smithii DSMZ 861	65.91	1.00

The MAG described in this work could provide valuable information regarding the ecology, metabolism, phylogeny, and evolution of the *M. paludis* clade.

### Data availability.

This whole-genome shotgun project has been deposited at DDBJ/ENA/GenBank under the version number JAIPRP000000000.1. The version described in this paper is version JAIPRP000000000.2. The BioProject accession number is PRJNA759916. The BioSample accession number is SAMN21209357. The draft shotgun metagenome assembly is available on https://figshare.com/ndownloader/files/28075893. The Hi-C reads and NextSeq data are available under accession numbers SRR11481801 and SRX6045636 to SRX6045639, respectively, in the SRA.
